# The Role of Extraversion, IQ and Contact in the Own-Ethnicity Face Recognition Bias

**DOI:** 10.3758/s13414-019-01947-6

**Published:** 2019-12-24

**Authors:** Peter J. Hills, Leanne Lowe, Brooke Hedges, Ana Rita Teixeira

**Affiliations:** Department of Psychology, Bournemouth University, Talbot Campus, Fern Barrow, Poole, BH12 5BB UK

**Keywords:** Face recognition, Intelligence, Individual differences, Extraversion, Own-ethnicity

## Abstract

While IQ is weakly related to the overall face recognition (Shakeshaft & Plomin, 2015), it plays a larger role in the processing of misaligned faces in the composite face task (Zhu et al., 2010). This type of stimuli are relatively novel and may reflect the involvement of intelligence in the processing of infrequently encountered faces, such as those of other-ethnicities. Extraversion is associated with increased eye contact which signifies less viewing of diagnostic features for Black faces. Using an old/new recognition paradigm, we found that IQ negatively correlated with the magnitude of the own-ethnicity bias (OEB) and that this relationship was moderated by contact with people from another ethnicity. We interpret these results in terms of IQ enhancing the ability to process novel stimuli by utilising multiple forms of coding. Extraversion was positively correlated with the OEB in White participants and negatively correlated with the OEB in Black participants suggesting that extraverts have lower attention to diagnostic facial features of Black faces, leading to poorer recognition of Black faces in both White and Black participants, thereby contributing to the relative OEB in these participants. The OEB is dependent on participant variables such as intelligence and extraversion.

The own-ethnicity bias (OEB) is the tendency for individuals of one ethnicity to recognise faces of their own ethnicity with greater efficiency (faster and with more accuracy) than faces of other ethnicities (Meissner & Brigham, 2001). While the effect is highly reliably and replicates across many ethnic groups (Ng & Lindsay, 1994), there is individual variability in the extent to which people display the OEB (Slone, Brigham, & Meissner, 2000). Contact with people from another ethnicity is one individual difference variable that has been found to affect the magnitude of the OEB (Chiroro & Valentine, 1995; Walker & Hewstone, 2006). However, there has not been a great deal of research exploring how other individual difference variables influence the OEB. This is despite highly methodologically similar studies producing conflicting results in OEB studies (such as the role of eye movements in the OEB, see Hills & Mahabeer, 2017, for a discussion). Here, we present a theoretical argument why extraversion and IQ might affect the OEB and test this experimentally.

Broadly speaking, there are two theories explaining the OEB: socio-cognitive (e.g., Sporer, 2001) and perceptual models (e.g., Valentine & Endo, 1992). Socio-cognitive models are based on the notion that faces are coded quickly as either own- or other-ethnicity (Levin, 1996). Own-ethnicity faces are processed using more effortful individuating coding whereas other-ethnicity faces are processed using categorisation processes (Levin, 2000). The notion is that motivation to process faces deeply is what drives the OEB. Motivation alone is insufficient to process other-ethnicity faces deeply: expertise is also required (Hugenberg, Young, Bernstein, & Sacco, 2010).

Perceptual expertise models of the OEB are based on the idea that when encoding or storing a face of the same ethnicity, individuals employ expert perceptual or cognitive mechanisms either based on configural/holistic processing (Michel, Rossion, Han, Chung & Caldara, 2006) or attentional allocation to diagnostic features (Hills & Lewis, 2006). Configural processing is a generic term involving three distinct processing types (Maurer, Le Grand, & Mondloch, 2002) of which holistic processing (where the features of the face and their relationships are processed in parallel as a gestalt, Rossion, 2008) has been linked to the OEB: Using the composite face task, participants employ more holistic processing for own-ethnicity faces than other ethnicity faces (Michel, Corneille & Rossion, 2007, 2010, but see Lewis & Hills, 2018). Holistic processing is based on our visual experience (Carey, de Schonen, & Ellis, 1992; Le Grand, Mondloch, Maurer, & Brent, 2001). The holistic processing account of the OEB is therefore that greater holistic processing is engaged for own-ethnicity faces than for other-ethnicity faces. Holistic processing is contrasted with the less expert featural processing, where facial features are processed in isolation.

While featural processing is not considered an expert method for encoding faces (but see Rhodes, Hayward & Winkler, 2006), there is evidence that certain facial features play a more important role in face recognition than others, and this is moderated by participant ethnicity. The eyes are the most diagnostic feature for White participants as revealed through: descriptions (Ellis, Deregowski, &Shepherd , 1975); deficits to recognition through concealment (Gosselin & Schyns, 2001); distortion detection (Hills, Marquardt, Young, & Goodenough, 2017); and eye movements (Althoff & Cohen, 1999; Arizpe, Kravitz, Yovel, & Baker, 2012; Hills & Pake, 2013). Black participants typically use different features when describing (Ellis et al., 1975) and viewing (Hills & Pake, 2013) faces focusing more on the nose and lips than White participants (White participants tend to describe the hair and eye colour more than Black participants). Indeed, directing attention away from the eyes and more to facial features described by Black participants increases recognition of Black faces (Hills & Lewis, 2011; Hills & Pake, 2013). This theoretical approach is different from the holistic/featural distinction account as it highlights that the attentional allocation paid to facial features is different depending on participant experience: The diagnostic features for discriminating between faces is also different for each ethnicity and when the attentional allocation matches the diagnostic features, recognition is greater than when it does not, leading to the OEB.

These models of the OEB indicate that there may be individual difference variables that may influence the magnitude of the effect in White participants. Taking first the diagnostic features hypothesis, any variable that moves encoding away from the eyes and more to features described by Black participants should reduce the OEB (for Black faces) in White participants. One such variable is extraversion. Extraversion is a personality type which is associated with sociability and sensitivity to reward, as well as positive emotions and high energy (Caspi, Roberts & Shiner, 2005). Extraverts engage in longer periods of eye contact than introverts or those scoring in the mid-range on extraversion scales (Kendon & Cook, 1969; Mobbs, 1968; Rutter, Morely & Graham, 1972). This suggests that introverts will make less eye contact than extraverts and therefore will look more at features described by Black participants. This may be the result of the experimental conditions or through years of experience where introverts look at other parts of the face rather than the eyes.

Given that there is correlation between the amount of eye fixation and face recognition accuracy in laboratory studies (Hills, Cooper, & Pake, 2013), we would suggest anything lowering the amount of eye fixation will reduce recognition performance for White faces. By the same logic, anything that increases attention to the diagnostic features of Black faces should increase the recognition of those faces. Indeed, when fixations are increased to the nose in Black faces, recognition performance increases (Hills & Pake, 2013). Therefore, we predict a positive correlation between extraversion and the OEB (for Black faces) in White participants because participants scoring higher in extraversion will not attend to features diagnostic for recognising Black faces and a negative correlation in Black participants for the same reason.

Returning to the socio-cognitive account and holistic accounts, we suggest that fluid intelligence, as measured by IQ can modulate the OEB. Fluid intelligence involves the ability to manipulate novel stimuli (Cattell, 1971) and is based on the notion of a general intelligence (the finding that there is a matrix of correlations between performance on a number of tasks, Spearman, 1927). While there is an overall positive manifold of abilities (Carroll, 1993), in which many cognitive abilities load onto the general intelligence factor, face recognition is relatively unique and is only moderately related to fluid intelligence (Connolly, Young, & Lewis, 2018; Gignac, Shankaralingam, Walker, & Kilpatrick, 2016; Shakeshaft & Plomin, 2015). Others indicate that face recognition is completely distinct from intelligence (Wilmer, Germine, & Nakayama, 2014), with a unique genetic influence (Shakeshaft & Plomin, 2015). When comparing the same participants, Zhu et al. (2010) did not find a correlation between IQ and the recognition of upright faces. However, they did find a correlation between IQ and the recognition of inverted faces and misaligned faces in the composite face effect. These types of stimuli are novel. Inverted faces, for example, are typically processed featurally (Friere, Lee, & Symons, 2000) and are viewed differently to upright faces (Barton, Radcliffe, Cherkasova, Edelman, & Intriligator, 2006). Participants with higher IQ should be able to cope with processing novel stimuli and therefore might engage the processing strategy that is optimal for the task (in this case more effective featural processing, or utilising holistic processing) to recognise facial stimuli they do not frequently encounter.

The argument presented in the preceding paragraph suggests that participants with higher IQ would be able to better recognise faces they have not frequently encountered. Indeed, there is plenty of evidence to suggest that those with higher IQ are able to recognise exemplars from various novel object categories (such as Greebles, Ziggerins, and Sheinbugs - three-dimensional computer-generated geometric shapes with additional features) and this ability is separate to the recognition of faces (Richler, Wilmer, & Gauthier, 2017). Based on the socio-cognitive and holistic accounts, one would expect a direct relationship between IQ and the OEB: Those with higher IQ will be able to process the novel faces better than those with lower IQ because of their ability to recognise novel visual categories rather than because they can employ strategies based on their ability to swap between strategies.

If the OEB is based on contact and experience (Cross, Cross, & Daley, 1971; Hancock & Rhodes, 2008; Walker & Hewstone, 2006), then participants' contact should moderate the relationship between IQ and the OEB. This is because contact allows people to gain experience of the features that are most useful in discriminating between faces of other-ethnicities. The implicit knowledge of what features to use to recognise other-ethnicity faces will be used to code faces by those who have high IQ because of improved flexibility in cognitive strategy used. In this way, the attentional allocation framework for the OEB indicates that there will be link between IQ and the OEB but it will be moderated by contact.

In this introduction, we have hypothesised two potential individual difference variables that might influence the OEB: Extraversion and IQ. To theorise why each might affect the OEB directly or moderated by experience, we have used the socio-cognitive, holistic processing, and attentional allocation frameworks. If the OEB is caused by participants not viewing the appropriate facial features, then extraversion should correlate with the OEB: extraverts are more likely to view the eyes and therefore less likely to view the diagnostic features for Black faces, thereby increasing the OEB in White participants. Further, IQ should directly correlate with the OEB if the OEB is due to socio-cognitive factors or holistic processing as those with higher IQ can utilise multiple techniques to recognise faces because of higher cognitive flexibility. However, if the OEB is related to extensive experience discriminating between faces of another ethnicity, thereby developing an understanding of the most diagnostic features, those with higher IQ should be able to recognise other-ethnicity faces more accurately than those with lower IQ. In other words, contact should moderate a relationship between IQ and the OEB. These hypotheses were tested in a correlational study involving the recognition of White and Black faces in a sample of White (Experiment 1) and Black participants (Experiment 2).

## Experiment 1

### Method

#### Participants

An opportunity sample of 231 (131 female, aged between 18 and 46 years, *M* = 21.59, *SD* = 5.77) people from the local area took part in this study. Sample size was determined by assuming a similar correlation between IQ and the OEB to that observed by Zhu et al. (2010) for IQ and inverted face recognition (*r* = .25, therefore required sample to achieve Power = .95 is *N* = 195, calculated using GPower 3.1). Participants volunteered following seeing an advert posted in a University participant recruitment site or on social media. Undergraduate students (*N* = 111) took part as part of a course requirement. Participants were recruited from the University and by social media. All participants self-reported they were ethnically White.

#### Design

A correlational design was employed with the predictor variables as extraversion and IQ and the primary criterion variable as the OEB. OEB was calculated according to the following equation:

1 $$ OEB=\left({d}_{own}^{\hbox{'}}-{d}_{other}^{\hbox{'}}\right)/\left({d}_{own}^{\hbox{'}}+{d}_{other}^{\hbox{'}}\right) $$

where *d'* is the stimulus discriminability as calculated using Signal Detection Theory (Green & Swets, 1966). This formula controls for the absolute lower performance for other-ethnicity faces (Hills & Lewis, 2006). Self-reported contact with other-ethnicity people was entered as a moderating variable. IQ was measured using Cattell's Culture Fair (Cattell & Cattell, 1960). Extraversion was measured using the International Personality Item Pool (IPIP) extraversion scale (Goldberg, 1999). Level of contact with Black individuals which was measured using a social contact questionnaire (Walker & Hewstone, 2006). We also recorded reaction times during learning and reaction times during test as well.

#### Materials

Cattell’s culture fair IQ test, Scale 2, Form A was used to measure IQ (Cattell & Cattell, 1960). This IQ test comprises of four tests of sequence completion, mental rotation, and novel problem-solving. This is a reliable and valid (Jordheim & Olsen, 1963) test of IQ with split-half reliability of between .90 and .92 (Knapp, 1960) and test-retest reliability of .85 (Cattell, 1968).

The IPIP extraversion scale (Goldberg, 1999) was used to determine participants’ level of extraversion. It consists of a series of 10 items relating to trait extraversion, such as “am skilled in handling social situations” and “feel comfortable around people” each measured on a 5-point Likert-type scale. Participants identify how much each statement relates to themselves. The anchor points were "Very inaccurate" (1) and "Very accurate" (5). This scale has a high reliability, α = .86 (Goldberg, 1999), *r* = .98 (Maples, Guan, Carter, & Miller, 2014), α = .88 (Ypofanti, Zisi, Zourbanos, Mouchtouri, Tzanne, Theordorakis, & Lyrakos, 2015).

A social contact questionnaire was used to ask participants about their social interactions with individuals of White and Black ethnicity (Walker & Hewstone, 2006). Example items include “I have a lot of friends who are of a Black ethnic origin” and “I spend a lot of time with people who are of a Black ethnic origin”. The same Likert-type scale was used here as for the IPIP. This test had moderately high reliability, Cronbach's α = .71.

The face recognition task was displayed on either a HP laptop with a 15.6-inch screen or on a Hewlett-Packard HP EliteDesk 800 G1 SFF, with a 24-inch wide-screen running OpenSesame Version 3.1.9 The experiment was completed in full screen with a resolution of 1024 by 768 pixels.

A total of 80 face identities were used from the Minear and Park (2004) database for the face recognition task. They were full frontal faces taken in front of a plain white background and were displayed in full colour in size 638 x 420 px and resolution 320 dpi. The faces did not contain any distinctive feature, jewelry, tattoos, or facial hair. Of these faces, 40 were ethnically Black (20 females) and 40 faces were ethnically White (20 females). The stimuli were aged between 18 and 32. Faces were either smiling or displayed a neutral expression. One image of each face was used during learning and one was used at test. This was counterbalanced across participants. Faces were also counterbalanced across-participants such that they appeared as a target face and as a distractor face an equal number of times. Our test was highly reliable with a Cronbach's α = .91 and a split-half reliability of *r* = .83.

#### Procedure

Participants were all tested individually in a quiet laboratory. After providing informed consent, participants completed Scale 2, Form A of the Cattell's Culture Fair (Cattell & Cattell, 1973) test. The test was administered in accordance with the instructions. Immediately following this, participants completed the face recognition task. This task consisted of three consecutive phases.

In the learning phase, participants were shown 40 (20 Black) faces sequentially in a random order. Each face was presented in the centre of the screen. Participants rated these faces on their distinctiveness ("How easy would this face be to spot in a crowd?") using the computer keyboard (Light, Kayra-Stuart & Hollander, 1979). The anchor points for this scale was 1 = "very difficult" and 9 = "very easy." Participants were encouraged to use the numbers in-between. The faces appeared on the screen until the participant responded. Following each face a random noise mask (size 256 x 256 px) was presented in the centre of the screen for 250 ms.

On completion of the learning phase, participants completed the IPIP (Goldberg, 1999) and the social contact questionnaire (Walker & Hewstone, 2006). Participants also provided demographic information. Participants responded using the computer keyboard. This task typically took four minutes. The test phase immediately followed this distractor phase.

In the test phase, participants were shown all 80 faces sequentially in a random order. Forty faces were old and 40 were new (half Black). Participants responded to each as to whether they had seen the face during the learning phase using the computer keyboard. Participants were instructed to be as fast and as accurate as possible. Each face was shown on the screen until the participant responded. Between each face, there was a random noise mask (size 256 x 256 px) presented in the centre of the screen for 250 ms. On completion of the last trial, participants were thanked and debriefed.

### Results

Participants old and new responses to the faces during the test phase were converted into the Signal Detection Theory (Green & Swets, 1966) measure *d'* using the Macmillan and Creelman (2005) method. With the number of stimuli used in this task, the maximum value for *d'* was 3.92, with 0 representing chance recognition performance. These were used to calculate the OEB using Formula 1. All questionnaire measures were calculated as per the original authors' instructions: the Extraversion scale was total score on the questionnaire; intelligence was the total raw score on the Cattell's Culture Fair Test; and the contact questionnaire was the total self-reported score. Descriptive statistics for each measure are presented in Table 1. Before running any analyses, we checked that our data met the appropriate assumptions. All assumptions were met.

**Table 1.** Descriptive statistics (mean, median, range) for Cattell's Culture Fair Test, the IPIP, the Social Contact Questionnaire, and the face recognition test for Experiments 1 (top row in each cell) and 2 (bottom row in each cell).

		Mean	Median	Range	Standard Deviation	Skewness	Kurtosis
Cattell's (Cattell & Cattell, 1973)		*33.57* *26.89*	*34* *28*	*20-42* *12-36*	*4.40* *4.75*	*0.91* *-1.00*	*-0.98* *1.75*
IPIP (Goldberg, 1999*)*		*72.85* *53.52*	*73* *53*	*38-97* *32-88*	*13.02* *15.39*	*-0.65* *0.52*	*-0.08* *-0.63*
Social Contact (Walker & Hewstone, 2005)	Black People	*8.53* *12.72*	*8* *14*	*3-15* *8-15*	*2.98* *2.55*	*0.38* *-0.51*	*-0.54* *-1.29*
	White People	*12.62* *7.61*	*12* *8*	*3-15* *3-15*	*2.31* *3.49*	*-1.17* *0.21*	*2.64* *-1.00*
Face recognition (e.g., Morgan & Hills, 2019*)*	Black faces	*1.85* *1.70*	*1.81* *1.70*	*0.25-3.92* *0.36-3.53*	*0.70* *0.70*	*0.32* *0.09*	*0.49* *-0.43*
	White faces	*2.24* *1.53*	*2.46* *1.52*	*-0.14-3.92* *-0.13-3.29*	*0.75* *0.76*	*-0.40* *0.44*	*0.40* *-0.20*
	Relative OEB	*0.09* *0.07*	*0.08* *0.07*	*-1.30-0.75* *-0.53-1.15*	*0.25* *0.31*	*-1.70* *0.67*	*10.80* *0.77*

Our first set of analyses verified that we obtained an OEB: Paired-samples *t*-tests revealed that recognition accuracy was greater for White faces (*M* = 2.24, *SD* = 0.75) than for Black faces (*M* = 1.86, *SD* = 0.70), *t*(230) = 8.47, *p* < .001, Cohen's *d* = 0.52. Reaction times during recognition were also longer for Black faces (*M* = 1224, *SD* = 427) than White faces (*M* = 1178, *SD* = 339), *t*(230) = 2.09, *p* = .038, Cohen's *d* = 0.12. Reaction times during encoding were not significantly longer for Black faces (*M* = 2854, *SD* = 1345) than White faces (*M* = 2772, *SD* = 1200), *t*(230) = 1.54, *p* = .125, Cohen's *d* = 0.06. We also found that our White participants demonstrated significantly more contact with White faces (*M* = 12.62, *SD* = 2.31) than Black faces (*M* = 8.53, *SD* = 2.98), *t*(230) = 16.35, *p* < .001, Cohen's *d* = 1.53.

**Figure 1.**
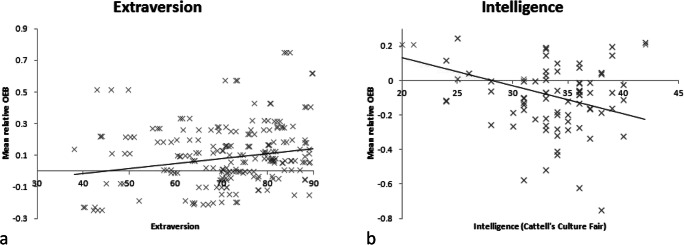
The relationship from Experiment 1 between the relative measure of the OEB and a. Extraversion and b. IQ.

Subsequently, we set out to test our first hypothesis - that extraversion would correlate positively with the OEB. Extraversion did correlate positively with the OEB, *r*(229) =.16, *p* = .013, shown in Figure 1a. Further correlations are specified in Table 2.

**Figure 2.**
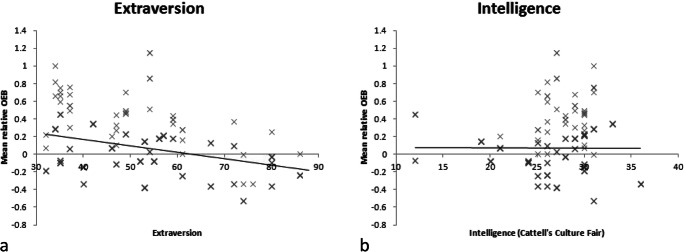
The relationship from Experiment 2 between the relative measure of the OEB and a. Extraversion and b. IQ.

**Table 2.** Correlation Coefficients for Extraversion and IQ and measures of face recognition for White participants, Experiment 1. We did not compute correlations between an OEB during encoding and Extraversion nor IQ because we did not find a significant OEB during encoding.

	Correlation with Extraversion	Correlation with IQ
Accuracy of Recognition (White faces)	***r*(229) = .01,*p*= .936**	***r*(229) = .14,*p*= .040**
Accuracy of Recognition (Black faces)	***r*(229) = -.12,*p*= .065**	***r*(229) =.27,*p*< .001**
OEB (Accuracy)	***r*(229) =.16,*p*= .013**	***r*(229) = -.29,*p*< .001**
RT of Recognition (White faces)	***r*(229) = -.01,*p*= .828**	***r*(229) = -.11,*p*= .108**
RT of Recognition (Black faces)	***r*(229) = -.08,*p*= .247**	***r*(229) = -.20,*p*= .002**
OEB (RT Recognition)	***r*(229) = -.10,*p*= .114**	***r*(229) = -.09,*p*= .192**
RT of encoding (White faces)	***r*(229) =.18,*p*= .005**	***r*(229) =.05,*p*= .465**
RT of encoding (Black faces)	***r*(229) =.14,*p*= .031**	***r*(229) = .00,*p*= .996**
Participant Age	***r*(229) = .05,*p*= .483**	***r*(229) = -.36,*p*< .001**
Relative Other-ethnicity contact	***r*(229) = -.05,*p*= .491**	***r*(229) = .15,*p*= .027**
Age and face-recognition: White Faces Black Faces OEB		***r*(229) = -.14,*p*= .039** ***r*(229) = -.07,*p*= .291** ***r*(229) = .09,*p*= .174**

Many of the correlations reported in this table are not independent of each other - for example, the OEB correlations are a combination of the correlations between Black and White faces and the DVs.

Because age is correlated with face recognition (Germine, Duchaine, & Nakayama, 2011, and as marginally shown in our data), we entered age into our further analyses in order to partial out the effects of it. We confirmed our second hypothesis - that IQ would correlate negatively with the OEB (age partialled out), *r*(228) = -.28, *p* < .001, shown in Figure 1b. Further correlations are specified in Table 2. Finally, we tested our third hypothesis that other-ethnicity contact would moderate the relationship between IQ and the OEB. In order to test this, we ran a hierarchical multiple linear regression with IQ and contact entered into the regression first, followed by the moderator (the interaction between IQ and contact) in step two. We centred the independent variables (by subtracting the mean from each value) before entering the data into the regression. Adding the moderator led to a significant change in *R*^2^ = .13, *F*(1, 227) = 40.43. Therefore, we report the overall model: It accounted for a significant proportion of the variance of the OEB, *R*^*2*^ = .50, *F*(3, 227) = 24.84, *p* < .001. Specifically, IQ correlated with the OEB IQ, *B* = -.18 (*CI*: -0.02, -0.00), *t* = 2.97, *p* = .003. Contact did not predict the OEB once the moderator was included, *B* = -0.08 (*CI*: -0.25, 0.04) *t* = 1.39, *p* = .165. Finally, and supporting our hypothesis the moderator uniquely predicted the OEB, *B* = 0.39 (*CI*: 0.06, 0.12), *t* = 6.36, *p* < .001. Including the moderator in the model reduced the size of the effect for both contact (*r*_0.1_ = -.17 to -.08) and IQ (*r*_0.1_ = -.26 to -.17).

### Discussion

In this study, we theorised that the OEB would be affected by participants' levels of extraversion. We found that there was a correlation between extraversion and the OEB in our White participants, indicating that with more extravert traits, the more the participant demonstrates an OEB. We interpret this based on the evidence that extraverts make significantly more eye contact than introverts (Kendon & Cook, 1969; Mobbs, 1968; Rutter, et al., 1972). Since the eyes are one of the least diagnostic features in Black faces (Shepherd & Deregowski, 1981), this means that extraverts would show poorer recognition of Black faces and greater recognition of White faces than introverts. The pattern we found was that the recognition of Black faces was affected by extraversion. White introverts, by avoiding eye contact, attend to more diagnostic features of Black faces and thereby reduces the magnitude of the OEB. This may be the result of years of experience avoiding the eyes or based purely on the task itself. The effect was observed only in accuracy and not in reaction time. During learning, we did find a positive correlation between extraversion and time spent encoding both Black and White faces. This, we interpret, represents extraverts' greater interest in people relative to introverts (McCrae, 1992).

We also theorised that IQ would correlate with the OEB, since participants higher in IQ are able to process novel stimuli more easily than participants lower in IQ. We found this correlation: IQ correlated with the ability to recognise Black and White faces, positively correlating with the recognition accuracy (and speed) of Black faces, thereby negatively correlating with the OEB. Given our interpretation of why the OEB is related to IQ, we anticipated that this link would be moderated by contact. This is precisely what was observed. Participants higher in IQ were more able to recognise other-ethnicity faces even with low contact, but those lower in IQ required more contact. These results are consistent with the notion that IQ allows participants to utilise different processing strategies to best accomplish the task at hand.

Together, these results can be interpreted within the attentional allocation framework of the OEB. Different facial features carry different diagnostic value for the recognition of White and Black faces (Ellis et al., 1975). Anything which forces participants to look at the diagnostic features of other-ethnicity faces will lead people to be better at recognising them. Participants higher in IQ might be better able to naturally look at the features that better discriminate between faces of other-ethnicities. This is because higher IQ allows for more flexible strategies to be used (Colzato, Van Wouwe, Lavender, & Hommel, 2006). When participants are given the instruction to individuate faces of other ethnicities, they are able to do so (Tanaka, Heptonstall, & Hagen, 2013; Young, Bernstein, & Hugenberg, 2010) thereby reducing the OEB (Baldwin, Keefer, Gravvelin, & Biernat, 2013; McGugin, Tanaka, Lebrecht, Tarr, & Gauthier, 2011). Such instructions are more effective for people who have significant other-ethnicity contact (Young & Hugenberg, 2012). Given these premises, we can surmise that participants higher in IQ are able to use this (potentially tacit) knowledge of the differential diagnosticity of features for each ethnicity of face to encode the features that are most diagnostic of the face they are presented with.

One could interpret these results within a holistic encoding framework if one accepts the idea that participants who have high IQ may be able to encode faces using both featural and holistic processing effectively. When presented with faces of their own-ethnicity, they employ the expert processing mechanisms associated with holistic processing. However, when processing faces of other ethnicities, they apply the appropriate featural coding more quickly and efficiently than those with low IQ. Evidence for this stems from eye-tracking evidence in which the high levels of individual variability in eye movement patterns (Peterson, & Eckstein, 2013) that leads to similar behavioural performance (Chuk, Chan, & Hsiao, 2017) or be unrelated to performance (Mehoudar, Arizpe, Baker, & Yovel, 2014). In other words, the face recognition system may well be somewhat flexible, with multiple strategies leading to successful performance. We suggest that participants high in IQ are better able to use different strategies and employ them appropriately.

There is a caveat regarding the findings of the present study. The study was conducted solely on a White sample. This was deliberate given the prediction about extraversion affecting face recognition: the eyes have not been reported as being critical for the recognition of faces of other ethnicities. The evidence regarding eye contact in extraverts stemmed from White samples. We do not know whether the same effect would replicate in non-White samples. Experiment 2 addressed this concern by testing a sample of Black participants.

## Experiment 2

The findings from Experiment 1 suggest that Extraversion and IQ separately predict the variability in the magnitude of the OEB in White participants. We hypothesised that extraversion leads to greater eye contact (and lesser coding of other facial features), which is detrimental to the recognition of Black faces. This explanation is irrespective of participant ethnicity, assuming Black extraverts also make more eye contact. This can be tested on a sample of Black participants, as conducted in Experiment 2. Based on the results from Experiment 1, the favoured explanation for the correlation between IQ and the OEB was that it would be moderated by contact: Those with higher IQ will be flexible in their strategy use and will therefore be able to view the most diagnostic features of the faces they are presented with because they have experience in understanding what are the diagnostic features. In order to rule out any stimulus effects (i.e., that the Black faces were somewhat atypical and this drove the correlation), testing a Black sample is further warranted.

### Method

An opportunity sample of 236 (125 female, aged between 18 and 52 years, *M* = 20.03, *SD* = 2.91) self-defined as Black people from the local area took part in this study (*N* = 82 were university students). Participants volunteered following seeing an advert posted in a university participant recruitment site or on social media.

The design, materials, and procedure were identical to those used in Experiment 1.

### Results

Our analytical structure for Experiment 2 replicated that of Experiment 1. Descriptive statistics are presented in Table 1. All assumptions for the relevant tests were met. Firstly we verified that we obtained an OEB: recognition accuracy was greater for Black faces (*M* = 1.70, *SD* = 0.69) than for White faces (*M* = 1.52, *SD* = 0.77), *t*(234) = 3.17, *p* = .002, Cohen's *d* = 0.25. Reaction times during encoding were significantly longer for White faces (*M* = 3297, *SD* = 2369) than Black faces (*M* = 2937, *SD* = 1964), *t*(234) = 2.79, *p* = .006, Cohen's *d* = 0.17. Reaction times during recognition were not significantly longer for White faces (*M* = 1224, *SD* = 427) than Black faces (*M* = 1178, *SD* = 339), *t*(234) = 0.69, *p* = .489, Cohen's *d* = 0.02. We also found that our Black participants demonstrated significantly more contact with Black faces (*M* = 12.75, *SD* = 2.55) than Black faces (*M* = 7.61, *SD* = 3.47), *t*(234) = 17.89, *p* < .001, Cohen's *d* = 1.69.

In Experiment 2, extraversion *negatively* (contrary to Experiment 1) correlated with the OEB, *r*(233) = -.36, *p* < .001. Further correlations are specified in Table 3. However, we did not find a significant correlation between IQ and the OEB (controlling for age), *r*(232) = -.01, *p* = .939. Additionally, we tested the hypothesis that other-ethnicity contact would moderate the relationship between IQ and the OEB with a hierarchical regression on the centred independent variables. The overall regression model (including IQ, contact, and the moderator - the interaction between IQ and contact) accounted for a significant proportion of the variance of the OEB, *R*^*2*^ = .09, *F*(3, 231) = 7.31, *p* < .001. This reflected a significant *R*^2^ change = *F*(1, 231) = 6.12, *p* = .014, when including the moderator. The moderator uniquely predicted the OEB, *B* = 0.06 (*CI*: 0.01, 0.11), *t* = 2.47, *p* = .014, whereas IQ did not, *B* = -0.01 (*CI*: -0.02, 0.00), *t* = 1.60, *p* = .112 and contact, *B* = 0.32 (*CI*: 0.16, 0.47), *t* = 3.97, *p* < .001.

**Table 3.** Correlation Coefficients for Extraversion and IQ and measures of face recognition for Black participants (Experiment 2). We did not compute correlations between an OEB during encoding and Extraversion nor IQ because we did not find a significant OEB during encoding.

	Correlation with Extraversion	Correlation with IQ
Accuracy of Recognition (White faces)	*r*(233) = .07, *p* = .324	*r*(233) = .08, *p* = .237
Accuracy of Recognition (Black faces)	*r*(233) = -.30, *p* < .001	*r*(233) = .03, *p* = .636
OEB (Accuracy)	*r*(233) = -.36, *p* < .001	*r*(233) = -.01, *p* = .899
RT of Recognition (White faces)	*r*(233) = .07, *p* = .324	*r*(233) = .08, *p* = .237
RT of Recognition (Black faces)	*r*(233) = -.13, *p* = .053	*r*(233) = -.22, *p* = .001
OEB (RT Recognition)	*r*(233) = -.25, *p* < .001	*r*(233) = -.03, *p* = .706
RT of encoding (White faces)	*r*(233) =-.21, *p* = .001	*r*(233) = -.05, *p* = .449
RT of encoding (Black faces)	*r*(233) = -22, *p* = .001	*r*(233) = .22, *p* = .001
Participant Age	*r*(233) = .20, *p* = .003	*r*(233) = .04, *p* = .553
Relative Other-ethnicity contact	*r*(233) = -.32, *p* < .001	*r*(233) = .03, *p* = .619
Age and face-recognition: White Faces Black Faces OEB		*r*(233) = .21, *p* = .001 *r*(233) = -.18, *p* = .005 *r*(233) = -.34, *p* < .001

Many of the correlations reported in this table are not independent of each other - for example, the OEB correlations are a combination of the correlations between Black and White faces and the DVs.

### Discussion

Experiment 2 replicated Experiment 1 in the crucial findings. Extraversion negatively correlated with the OEB in Experiment 2 rather than positively in Experiment 1. This is consistent with our predictions based on the evidence that extraverts tend to make more eye contact than introverts (Kendon & Cook, 1969; Mobbs, 1968; Rutter, et al., 1972) and that the eyes do not help discriminate between Black faces as well as other features do (Shepherd & Deregowski, 1981). Therefore, extraversion causes an enlarged OEB in White participants and a reversed OEB in Black participants as shown by the present data. In both experiments, this effect was driven by extraversion correlating with recognition performance of Black faces rather than White faces. In order words, extraversion does not affect the recognition performance of White faces.

The previous point is important theoretically as one could have assumed that extraversion would enhance the recognition of White faces because it would increase eye contact to the highly discriminative eyes. However, since we did not find this correlation, we suggest that eye contact is not increasing the recognition of White faces as has been found by previous authors (Hills & Lewis, 2011). There are two plausible explanations for this effect. Firstly, eye fixation as caused by extraversion may not be related to encoding them in an effortful manner. There is evidence to suggest that extraverts do engage in more shallow processing (Morgan & Hills, 2019) which fits with this notion. Alternatively, the impact extraversion is having is on the relative encoding of the other features: Attending to features other than the eyes is more beneficial for discriminating between Black faces and by increasing this (or participants who do this more) improves the recognition of such faces. We believe that this hypothesis is more plausible because of our participant sample were all British. This means that they were more aware of British cultural norms (including that avoiding eye contact is considered rude). Potentially, this means our participants were more likely to look at the eyes more than Black participants from more predominantly Black countries (3.3% of the population of Britain are ethnically Black, ONS, 2018). This might mean that, while Black participants typically utilise features other than the eyes to discriminate between faces, those in Britain may not as much as those from more predominantly Black countries. This would require cross-cultural eye tracking evidence to address this point.

Experiments 1 and 2 were also consistent in finding the moderation of the relationship between IQ and the OEB by contact. This result is entirely consistent with the notion that participants higher in IQ are able to use knowledge of the differential diagnosticity of features for each ethnicity of face to encode the features that are most diagnostic of the face they are presented with. This relationship is, therefore, due to flexible use of cognitive strategies based on knowledge of the diagnostic visual features of encountered faces, consistent with our discussion about extraversion. Overall, these results support the attentional allocation framework of the OEB.

At this point, we must discuss some inconsistent findings across Experiments and with previous published work. While none of these inconsistent findings actually affect the theoretical interpretation of the results, it is worth exploring them for a complete picture of our results. In Experiment 1, we found a negative correlation between own-ethnicity face recognition and age and in Experiment 2 we found a positive correlation between other-ethnicity face recognition and age. These results are inconsistent with Germine et al.'s (2011) findings that age should positively correlate with (own-ethnicity) face recognition. We explain these findings by highlighting that our sample was very limited in age range (with a majority of participants around the age of 21 years). This was to match the face sample we were using to avoid the own-age bias (Anastasi & Rhodes, 2005). It is, therefore, not a valid sample to explore the effects of age. It is highly likely that our results here are the result of such a limited sample and do not represent a reliable effect.

In Experiment 1, we found that IQ correlated with participant age and participant relative other-ethnicity contact. In Experiment 2, these correlations were not present. This likely reflects the fact that the two samples were not matched for IQ. The sample in Experiment 2 had lower average IQ and extraversion than those in Experiment 1 (hence why we tested this across two Experiments). This did not drive the effect, however, as the correlation between age and IQ was not significantly larger in the sample scoring higher than 36 on the Cattell's Culture Fair test, *r*(95) = -.39, *p* < .001. The correlation between contact and IQ might be driven by socio-cultural effects not considered: For Black participants it is harder to avoid the majority White people - therefore Black people are more likely to have every day experience with the majority group. Therefore, the contact correlation is less likely to be present. However, for the White participants the correlation between IQ and other-ethnicity contact may reflect that those with higher IQ seek out more novel experiences than those with lower IQ. Since this issue goes beyond the current thesis and does not affect the overall argument we do not consider it further.

## General discussion

Two experiments found a correlation between IQ and the OEB moderated by contact. This is consistent with the thesis that those with higher IQ are implicitly aware of the most diagnostic features that assist in discriminating between faces of another ethnicity and are better able to use these in recognition tasks than those with lower IQ. The lack of a direct correlation between IQ and the OEB indicates that contact is required for those with higher IQ to utilise different processing mechanisms suggesting that something about the experience teaches participants to better code other-ethnicity faces.

These results can go someway to help explain inconsistencies in the research literature. For example, Arizpe, et al. (2016) reviewed literature on eye movements and their role in the OEB. They highlighted how there have been contradictory reports published in the literature despite using similar methods. They indicated that analytical differences may have impacted on such findings. Here, we indicate that participant individual variability may cause contradictory findings: If a study was conducted solely on participants of higher IQ, then the present results indicate that they may show a smaller OEB and may even show differential viewing and processing patterns for faces of their own versus another ethnicity. This is particularly problematic for studies using small samples. Further, given that many psychological studies explore participants from university, they are likely to be of higher IQ than those who have not attended university. It may be that some of the effects discovered in student samples may not replicate to other samples. Further, the results (especially for extraversion) reported here are likely to reflect only the ethnicities tested in the present Experiment. Further work would need to be conducted to see if the current findings would replicate in Asian, for example, samples.

While we have found consistent results here, we must consider limitations of using a measure of IQ. We have already indicated that university students are likely to have higher IQ than non-university students. This highlights a clear confound between IQ and numerous other factors that could have influenced our results. While IQ is supposed to correlate with a wide variety of cognitive factors (Carroll, 1993), this leads to the possibility that other factors might be contributing to the relationship between IQ and the OEB. For example, socio-economic status (which may lead to educational and experiential opportunities), schooling (including test experience), and potentially motivation. Those with higher IQ might simply be more able to cope with the task demands and be familiar with the testing method. These cannot be excluded from the present discussion but could offer avenues for further research into the individual differences associated with the OEB.

In summary, we found that extraversion, IQ, and other-ethnicity contact correlated with the OEB. Specifically, White extraverts are more likely to show an OEB than introverts while the converse was true for Black extraverts, potentially due to increased eye contact leading to lower encoding of features diagnostic to the recognition of Black faces. Participants with higher IQ showed a smaller OEB. This effect was moderated by contact. Such results indicate that participants with higher IQ are able to use more flexible processing systems allowing them to process other-ethnicity faces better than participants with lower IQ.

### Open practices statement

The data reported here is available on BORDAR: http://bordar.bournemouth.ac.uk/95/ . None of the experiments were preregistered.
